# Ovarian Endometrioma Disrupts Oocyte‐Cumulus Communication and Mitochondrial Function, With Melatonin Mitigating the Effects

**DOI:** 10.1111/cpr.13800

**Published:** 2025-01-21

**Authors:** Lei Ge, Yali Yang, Yuqing Gao, Tianxia Xiao, Wakam Chang, Hefei Wang, Zhonglin Xiao, Jie Chen, Mengxia Li, Ming Yu, Ping Jin, Jian V. Zhang

**Affiliations:** ^1^ Center for Energy Metabolism and Reproduction Shenzhen Institute of Advanced Technology, Chinese Academy of Sciences Shenzhen Guangdong China; ^2^ University of Chinese Academy of Sciences Beijing China; ^3^ Shenzhen Key Laboratory of Metabolic Health Shenzhen Guangdong China; ^4^ Department of Biomedical Sciences, Faculty of Health Sciences University of Macau Macau China; ^5^ Faculty of Data Science City University of Macau Macau China; ^6^ Shenzhen Maternity and Child Healthcare Hospital Shenzhen Guangdong China; ^7^ The First School of Clinical Medicine Southern Medical University Shenzhen Guangdong China; ^8^ Faculty of Pharmaceutical Sciences Shenzhen University of Advanced Technology Shenzhen Guangdong China; ^9^ Sino‐European Center of Biomedicine and Health Shenzhen Guangdong China

**Keywords:** melatonin, oocytes quality, ovarian endometriosis, ovarian infertility, reactive oxygen species, Transzonal projections

## Abstract

Ovarian endometrioma (OEM), a particularly severe form of endometriosis, is an oestrogen‐dependent condition often associated with pain and infertility. The mechanisms by which OEM impairs fertility, particularly through its direct impact on oocyte‐cumulus cell (CC) communication and related pathways, remain poorly understood. This study investigates the impact of OEM on oocyte‐CC communication and explores melatonin's therapeutic potential. We used a mouse model of OEM and employed ovarian transcriptome and gene set enrichment analyses to identify disrupted gene pathways, alongside phalloidin staining for cytoskeletal analysis, gap junction coupling analysis for intercellular communication, and mitochondrial function assessments for cellular metabolism. Our results showed that OEM significantly impairs steroidogenesis and cumulus cell function, leading to increased apoptosis, disrupted transzonal projections (TZPs), and impaired antioxidant transfer to oocytes. This culminates in oxidative stress, mitochondrial dysfunction, and compromised ATP production. OEM oocytes also exhibited severe abnormalities, including DNA damage, maturation defects, spindle assembly disruptions, and increased aneuploidy. This study identifies disrupted TZPs as a key pathological feature in OEM and highlights melatonin's potential to restore intercellular communication, mitigate oxidative damage, and improve reproductive outcomes.

## Graphical abstract

Diagram illustrating the impact of OEM on oocyte‐cumulus communication, showing reduced TZPs, mitochondrial dysfunction, increased oxidative stress, DNA damage, and apoptosis. Melatonin (MLT) treatment improves communication, enhances mitochondrial function, reduces DNA damage, and restores oocyte quality, offering insights into potential therapeutic mechanisms for OEM‐related oocyte damage.

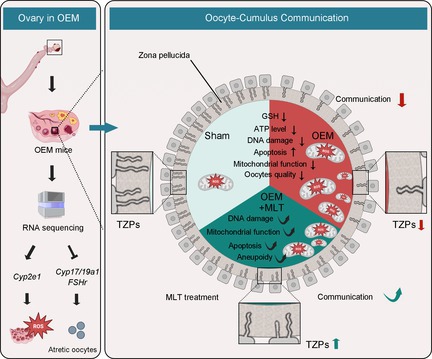

## 1 Introduction

Endometriosis (EM) is a prevalent oestrogen‐dependent gynecologic disorder affecting approximately 50% of infertile women of reproductive age [1]. Amongst its subtypes, ovarian endometriosis (OEM) represents the most severe form, affecting up to 44% of cases and significantly impacting ovarian function, including oocyte development and hormone secretion [2, 3, 4]. This frequently results in fewer antral follicles, increased oxidative stress in granulosa cells, and lower fertilisation and implantation rates, which are key contributors to EM‐related infertility [5, 6, 7]. Notably, patients undergoing Assisted Reproductive Technology (ART) with donor eggs exhibit normal embryo survival and implantation potential, emphasising the critical role of oocyte quality and the follicular microenvironment in EM‐related infertility [4, 8]. However, the precise mechanisms by which EM impairs oocyte quality and follicular function remain poorly understood.

Ovarian follicle development depends on the coordinated processes of somatic cell proliferation, differentiation, and oocyte maturation [9, 10]. Cumulus cells (CCs), which surround and nourish oocytes, are essential to these processes. The bidirectional communication between oocyte and CCs is crucial for granulosa cell function and the production of viable oocytes [11], a process mediated primarily by transzonal projections (TZPs)‐specialised filopodia that connects oocytes to adjacent somatic cells [12]. Effective oocyte‐CC coordination is crucial for successful ART outcomes, as proper communication supports oocyte maturation and pregnancy rates [13, 14]. Disruption of these projections may therefore lead to compromised oocyte quality, contributing to the subfertility observed in OEM patients.

Research on OEM's reproductive impact has primarily focused on granulosa cell abnormalities and oocyte quality [3, 12, 15]. Ferrero et al. identified disruptions in steroid metabolism and oxidative stress using single cell sequencing of oocytes from OEM patients [16]. Additionally, exposure to OEM follicular or peritoneal fluid can induce oocyte maturation arrest in animal models [15, 17, 18]. Granulosa cells from OEM patients often display a senescent phenotype and heightened oxidative stress [12, 19]. Despite this, how OEM disrupts the communication between oocytes and CCs, particularly through TZPs, remains unclear.

Disrupted circadian rhythms have been linked to a higher prevalence of EM [20], suggesting a potential role for melatonin (MLT), a regulator of circadian cycles, in modulating EM progression and reproductive outcomes [21]. In addition to its circadian regulatory functions, MLT's potent antioxidant properties have been shown to mitigate the oxidative stress in the ovarian environment of OEM patients [12, 22, 23]. Clinical trials suggest that MLT supplementation improve fertilisation and pregnancy rates in ART patients [24, 25]. However, further investigation is required to explore MLT's potential to improve oocyte quality and restore the follicular microenvironment in EM patients.

In this study, we assessed the integrity of TZPs in OEM and their effects on oocyte quality, as well as the impact of MLT administration. Using a mouse model of OEM, we observed increased apoptosis in CCs and disrupted oocyte‐CC communication, resulting in impaired oocyte quality, chromosomal aneuploidy, and spindle assembly defects. Importantly, MLT enhanced oocyte quality by improving oocyte‐CC communication and promoting oocyte maturation. This study hypothesizes that TZP disruption is a key mechanism by which OEM impairs oocyte quality, a previously unrecognised aspect of this condition's pathology, and that MLT may restore this critical communication, thereby improving reproductive outcomes.

## 2 Materials and Methods

### 2.1 Animal Experiments

Female C57BL/6J mice (8 weeks old) were purchased from Guangdong Medical Laboratory Animal Center (Guangdong, China). Animal care followed the Guide for the Care and Use of Laboratory Animals in Guangdong Province and was approved by the Ethics Committee at Shenzhen Institutes of Advanced Technology (Approval Number: SIAT‐IACUC‐200313‐YYS‐YM‐A1105). We used a murine OEM model based on Hayashi's method to evaluate ovarian dysfunction and follicular development [26]. Mice were acclimatised for 1 week at 23°C–25°C with a 12‐h dark/light cycle with water provided in a pathogen‐free condition.

### 2.2 RNA Extraction and mRNA Expression Analysis

Total RNA was extracted from ovarian tissues using TRIzol (Takara, China) for RNA‐seq (three replicates per group) and sent to OE Biotechnology LTD for sequencing. Gene expression was quantified using the FPKM method, with differential expression set at *p* ≤ 0.05 and |Log2 (fold change)| ≥ 2. KEGG pathway analysis and Gene Set Enrichment Analysis (GSEA) were performed using publicly available databases.

### 2.3 Oocyte and Cumulus‐Oocyte Complex (COC) Collection

Mice received 5 IU of PMSG (Ningbo San Sheng Biotech, China), and mature oocytes were collected 44 h later via ovarian puncture. Germinal vesicle (GV)‐stage oocytes and COCs were isolated from antral follicles and cultured in M16 medium (M7292, Sigma‐Aldrich, USA) at 37 °C in 5% CO2. For recovery experiment, OEM mice were randomly divided into OEM and OEM + MLT groups, with the latter receiving melatonin (MLT) (30 mg/kg) every 12 h following PMSG injection. The OEM group received saline. In vitro, oocytes and COCs were cultured in M16 medium with MLT (1 × 10^−7^ mol/L).

### 2.4 Staining and Analysis of TZPs

Oocytes were fixed in 3.7% PFA for 50 min, blocked with 1% BSA, and incubated overnight at 4°C with Actin‐Tracker Red‐555 (1:200, C2203S, Beyotime Biotech). After washing, oocytes were counterstained with Hoechst 33342 (1:100, 14,533, Sigma) and mounted using SlowFade Gold (Life Technologies), and visualised with a confocal microscope (Leica SP8). Fluorescence was quantified using Image‐J software (NIH, Bethesda, MD).

### 2.5 Immunofluorescence

Oocytes were fixed in 3.7% PFA, blocked, and incubated overnight with primary antibodies at 4°C, including FITC‐α‐tubulin (1:500, F2168, Sigma Aldrich), and Actin‐Tracker Red‐555 (1:200, C2203S, Beyotime Biotech). After washing, oocytes were counterstained with Hoechst 33342 (1:100, 14,533, Sigma) for 10 min, mounted, and examined with a confocal microscope (Leica sp8). Fluorescence was semi‐quantitatively analysed using NIH Image programme ImageJ software.

### 2.6 Gap Junctional Coupling Analysis

Gap junctions between oocytes and CCs were assessed using calcein‐AM staining. COCs were incubated with 1 mM calcein‐AM for 15 min and observed under a fluorescence microscope to evaluate dye transfer from CCs to oocytes. The intensity ratio was calculated using ImageJ software.

### 2.7 Mitochondrial Function Measurement

ATP levels in GV‐stage oocytes were measured using an ATP Assay Kit (S0027, Beyotime Biotech). Briefly, GV oocytes were denuded of their zona pellucida in Tyrode's solution (T1788, Sigma‐Aldrich), lysed with 20 μL of lysis solution on ice for 5  min, and then incubated with 100 μL ATP assay solution for 10  min. Luminescence was measured using a luminometer (POLARstar Omega, USA). Mitochondrial membrane potential (MMP) was determined using JC‐1 dye (C2006, Beyotime Biotech). Briefly, oocytes were incubated in the M2 medium with JC‐1 for 30 min, washed, and analysed for JC‐1 aggregates and monomers using a fluorescence microscope (Leica sp8). The ratio of aggregates to monomers was calculated to evaluate MMP. For mitochondrial distribution analyses, oocytes were incubated in an M2 medium with 500 nM MitoTracker Green kit (M7514, Thermo Fisher Scientific, USA) for 30 min. Fluorescence was analysed under a confocal microscope (Leica sp8).

### 2.8 Monitoring of GSH/ROS Levels in Oocytes

Reactive oxygen species (ROS) and glutathione (GSH) levels in oocytes were detected by 10 mM oxidation sensitive fluorescent probe dichlorofluorescein (DCFH) (S0033M, Beyotime Biotech) and 50 μM of Monochlorobimane (mBCL) (69,899, Sigma‐Aldrich), respectively, for 30 min. Oocytes were then washed and visualised using a confocal microscope (Leica sp8).

### 2.9 Apoptosis Detection

Apoptosis in COCs and oocytes were stained with an Annexin‐V Staining Kit (C1062M, Beyotime Biotech). Briefly, samples were stained with 95 μL of binding buffer containing 5 μL of Annexin‐V‐FITC for 20 min, washed, and imaged using a confocal microscope (Leica sp8).

### 2.10 Statistical Analysis

All experiments were repeated at least three times. Statistical comparisons between two groups were performed using unpaired *t*‐tests with Prism 9 software (GraphPad, USA), following confirmation of normality with the Anderson–Darling and Kolmogorov–Smirnov tests. For multiple‐group comparisons, one‐way ANOVA followed by Tukey's post hoc test was used. Data are presented as means ± SD. Statistical significance was set at * (*p* < 0.05), ** (*p* < 0.01), *** (*p* < 0.001), and **** (*p* < 0.0001). Not significant (ns) at *p* ≥ 0.05.

## 3 Results

### 3.1 Transcriptome Analysis Reveals Disruptions in Hormone Production, Oxidative Stress, and Cellular Communication in OEM

**FIGURE 1:**
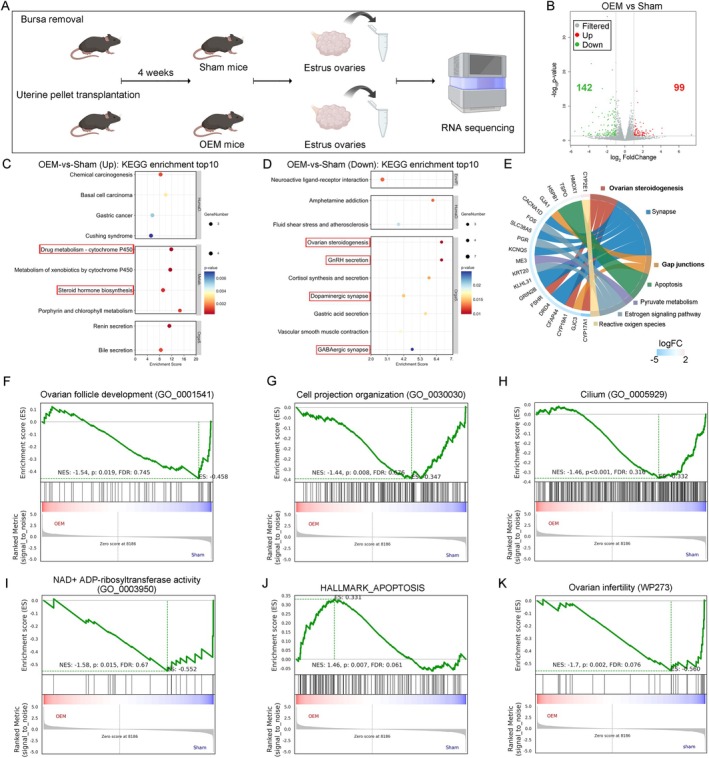
Molecular insights into ovarian damage in OEM mice. (A) Experimental strategy for assessing ovarian damage using Bulk RNA sequencing in individual ovaries after 4 weeks. (B) Scatter plot comparing transcriptomes of Sham‐operated and OEM ovaries, highlighting transcripts with a fold‐change greater than two (red, upregulated; green, downregulated) in OEM ovaries. (C, D) KEGG pathway analysis showing the top 10 upregulated and downregulated pathways in OEM ovaries, with respective enrichment scores. (E) Chord diagram illustrating the expression patterns of differentially expressed genes (DEGs) identified in OEM ovaries. (F–K) Gene Set Enrichment Analysis (GSEA) demonstrating gene enrichment in OEM ovaries within the following pathways: “Ovarian follicle development”, “Cell projection organization”, “Cilium”, “NAD+ ADP‐ribosyl transferase activity”, “Apoptosis”, and “Ovarian infertility”.

To elucidate the molecular mechanisms underlying follicle and oocyte damage in ovarian endometriosis (OEM), we performed bulk RNA sequencing (RNA‐seq) on ovaries from three estrus‐stage mice per group, followed by bioinformatics analysis (Figure 1A). In OEM mice, we identified 99 upregulated and 142 downregulated genes compared to Sham controls (Figure 1B). KEGG pathway analysis showed enrichment in genes related to primordial follicle activation and upregulation of mTOR signalling, consistent with clinical observations of OEM [4, 27]. Downregulated genes were predominantly involved in ovarian steroidogenesis, oestrogen signalling, and synaptic gap junction pathways (Figure 1C,D).

The chord diagram illustrated the expression patterns of these differentially expressed genes (DEGs) (Figure 1E). Notably, upregulation of genes like *Cyp2e1* was linked to reactive oxygen species (ROS) pathways, indicating heightened oxidative stress in OEM ovaries [28, 29, 30]. Conversely, downregulation of genes associated with ovarian steroidogenesis (e.g., CYP17A1, CYP19A1) and oestrogen signalling pathways suggests impaired hormone production. Reduced expression of synapse‐related and gap junction genes (e.g., FSHr, GRIN2B) indicates disruptions in cellular communication and neural regulation.

Gene set enrichment analysis (GSEA) showed downregulation in “ovarian follicle development” (ES = −0.458), indicating impaired folliculogenesis (Figure 1F). The downregulation of “cell projection organization” genes (ES = −0.347) suggest compromised cell junction integrity (Figure 1G), and negative enrichment of the “Cilium” gene set (ES = −0.332) points to potential disruptions in ciliary function (Figure 1H). Decreased expression of genes linked to “NAD+ ADP‐ribosyltransferase activity” suggests diminished cellular repair and stress response (Figure 1I). The “Hallmark Apoptosis” gene set was upregulated (ES = 0.331), indicating increased apoptosis in OEM tissues (Figure 1J). Lastly, significant downregulation of the “Ovarian Infertility” gene set (ES = −0.560) underscores the link between OEM and impaired fertility (Figure 1K). These findings highlight molecular disruptions in hormone production, oxidative stress response, and cellular communication, contributing to ovarian dysfunction and reduced fertility in OEM.

### 3.2 OEM Induces Apoptosis and Impairs Cumulus‐Oocyte Communication

**FIGURE 2:**
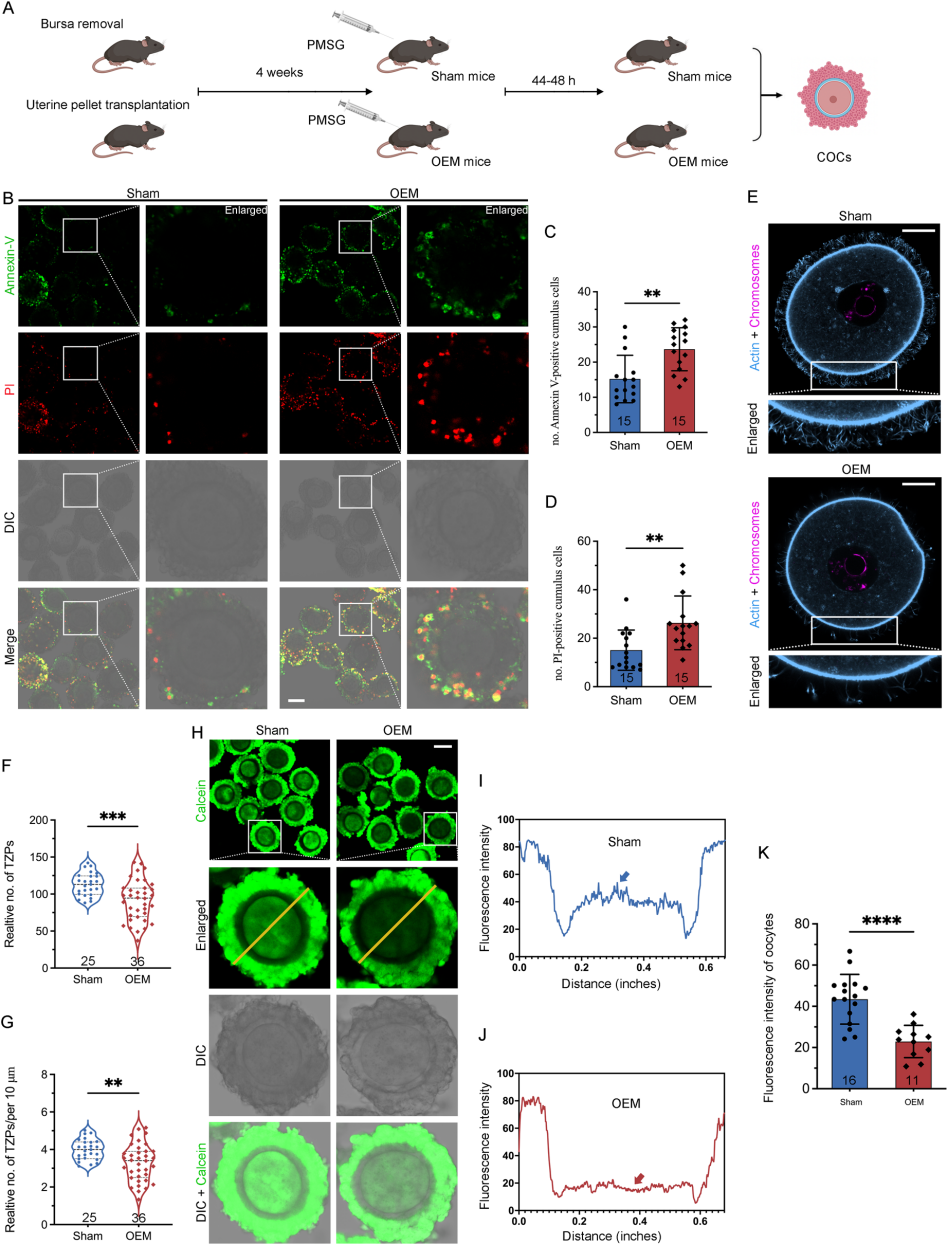
Cumulus cell apoptosis and impaired TZP generation in OEM mice. (A) Experimental timeline depicting the OEM mouse model and hormonal superovulation protocol. (B) Representative images of Annexin V (green) and Propidium Iodide (PI) (red) staining in cumulus‐oocyte complexes (COCs) from Sham and OEM mice (Scale bar, 100 μm). (C, D) Quantification of Annexin V‐positive and PI‐positive cumulus cells (CCs) from confocal sections. Sham oocytes, *n* = 15; OEM oocytes, *n* = 15. (E) Confocal images of COCs from Sham and OEM mice showing TZPs (blue) labelled with phalloidin. Lower panels show magnified views, and the oocyte nucleus outlined in magenta. (F, G) Quantification of TZP number and density from equatorial confocal sections. Sham oocytes, *n* = 25; OEM oocytes, *n* = 36. (H) Representative images of Calcein dye transmission from CCs to oocytes (Scale bar, 100 μm), and (I, J) corresponding fluorescence intensity distributions in OEM and Sham mice. (K) Integrated optical density (IOD) measurement in oocytes reflecting gap junction permeability. Sham oocytes, *n* = 16; OEM oocytes, *n* = 11. Statistical significance was set at *p < 0.05, **p < 0.01, ***p < 0.001, and ****p < 0.0001. Not significant (ns) at *p* ≥ 0.05.

Previous studies have shown that oxidative stress and apoptosis are elevated in granulosa cells of EM patients. To determine whether OEM similarly affects cumulus cells (CCs), specialised granulosa cells supporting oocytes, we assessed CC functions under OEM conditions. Mice underwent PMSG‐induced superovulation 4 weeks post‐surgery (Figure 2A), and cumulus‐oocyte complexes (COCs) were collected 44–46 h later for Annexin‐V (AV)/Propidium Iodide (PI) apoptosis assays (Figure 2B). The number of AV and PI‐positive CCs in OEM mice significantly increased compared to the Sham‐operated controls, suggesting elevated early and overall apoptosis in CCs (Figure 2C,D).

Transzonal projections (TZPs), crucial for oocyte development and energy exchange, extend from CCs to oocytes [31]. We stained COCs with F‐actin using phalloidin to evaluate TZP integrity (Figure 2E). OEM mice showed a reduction in TZP number and density (Figure 2F,G), indicating compromised oocyte‐CC connectivity. This was further supported by decreased expression of the FSHr, which is crucial for maintaining TZP stability (Figure 1E), as FSH regulates TZP dynamics.

To further evaluate gap junction‐mediated oocyte‐CC communication, we incubated COCs with calcein‐AM (Ca‐AM), a fluorescent marker transferred through gap junctions (Figure 2H). Sham‐operated mice displayed strong fluorescence, indicating intact communication (Figure 2I). In contrast, OEM COCs exhibited significantly reduced fluorescence (Figure 2J,K), reflecting impaired intercellular signalling. These findings suggest that OEM increases apoptosis and disrupts communication between oocytes and CCs, potentially compromising oocyte maturation and function.

### 3.3 OEM Impairs Cumulus‐Oocyte Complex Morphology and Oocyte Function Through Oxidative Stress and Mitochondrial Dysfunction

**FIGURE 3:**
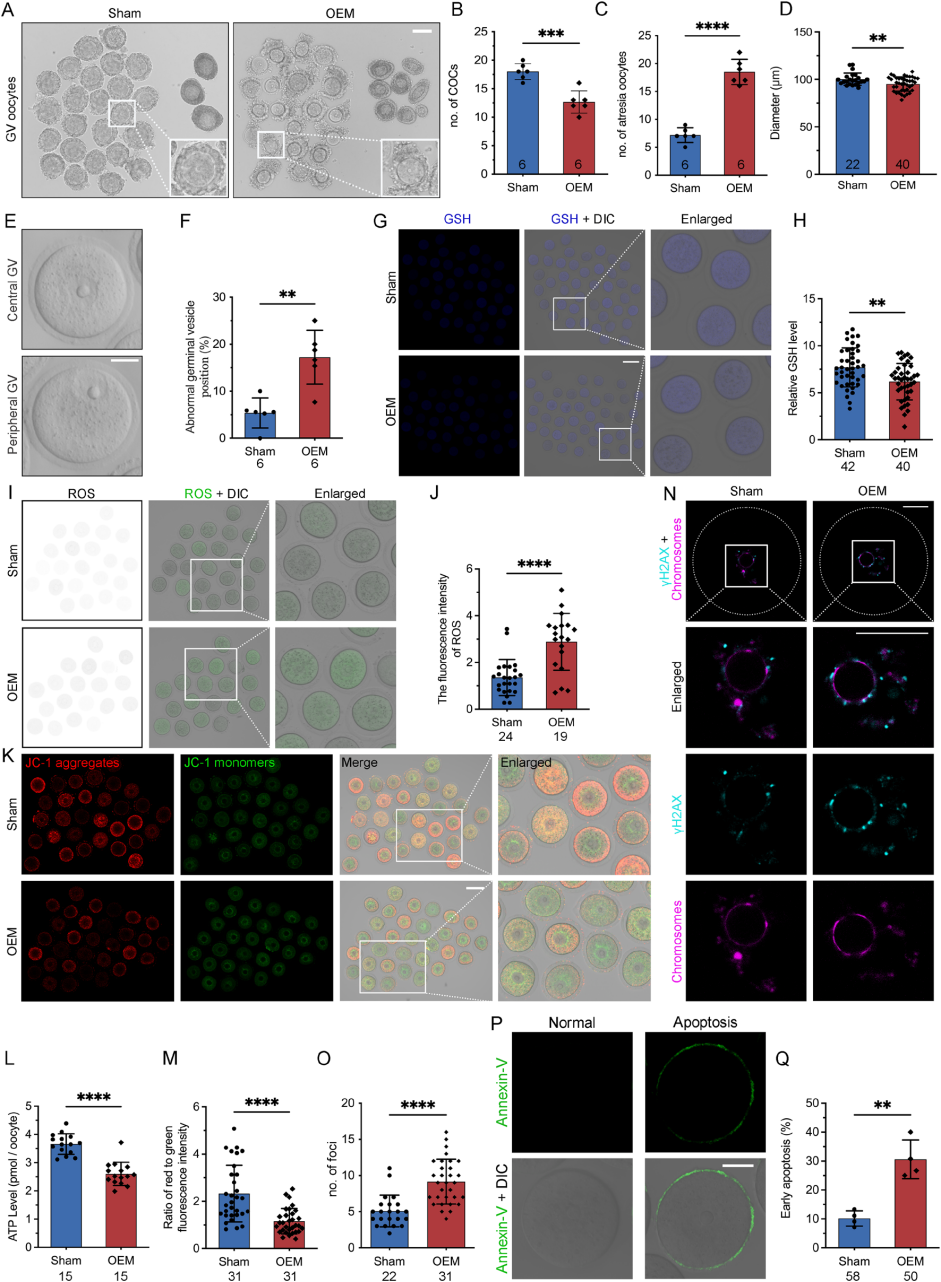
Morphological and functional changes in oocytes from OEM mice. (A) Representative images of cumulus‐oocyte complexes (COCs) and atretic oocytes (Scale bar, 120 μm). (B, C) Quantification of COCs and atretic oocytes in Sham and OEM mice. Sham mice, *n* = 6; OEM mice, *n* = 6. (D) Measurement of germinal vesicle (GV) oocyte diameter from Sham and OEM mice. Sham oocytes, *n* = 22; OEM oocytes, *n* = 40. (E, F) Representative images of central and peripheral GV and the abnormal position rate in Sham and OEM oocytes. Sham mice, *n* = 6; OEM mice, *n* = 6. (G, H) Glutathione (GSH) levels and corresponding intensity ratios in Sham and OEM oocytes. Sham oocytes, *n* = 42; OEM oocytes, *n* = 40. (I, J) DCFHDA staining for reactive oxygen species (ROS) levels in Sham and OEM oocytes (Scale bar, 100 μm), with quantification of fluorescence intensity. Sham oocytes, *n* = 24; OEM oocytes, *n* = 19. (K) Representative images show the JC‐1 aggregates (red) and monomers (green) in mouse oocytes from Sham and OEM. (L) Scatter plots illustrate the ATP levels of mouse GV oocytes. Sham oocytes, *n* = 31; OEM oocytes, *n* = 31. (M) Bar graph displaying the JC‐1 aggregate‐to‐monomer ratio. Sham oocytes, *n* = 15; OEM oocytes, *n* = 15. (N, O) γ‐H2AX staining and quantification of DNA damage. Sham oocytes, *n* = 22; OEM oocytes, *n* = 31. (P, Q) Annexin V staining for apoptosis and statistical results in Sham and OEM groups. Sham oocytes, *n* = 58; OEM oocytes, *n* = 50. Statistical significance was set at *p < 0.05, **p < 0.01, ***p < 0.001, and ****p < 0.0001. Not significant (ns) at *p* ≥ 0.05.

To evaluate the impact of OEM on oocyte quality, we examined the number and morphology of cumulus‐oocyte complexes (COCs) and atretic oocytes (Figure 3A). OEM mice exhibited a significantly lower count of normal COCs (12.67 ± 1.966) compared to the Sham group (18.00 ± 1.414) (Figure 3B), while the number of atretic oocytes was threefold higher in OEM (18.50 ± 2.258) versus Sham group (7.167 ± 1.329) (Figure 3C). Examination of oocyte diameter by removing CCs revealed a significant reduction in OEM mice (Figure 3D), indicating restricted oocyte growth, consistent with clinical findings in EM [32]. Additionally, a higher proportion of germinal vesicle (GV)‐stage oocytes in OEM mice displayed deviated morphology, suggesting disrupted microfilament stabilisation and oocyte maturation (Figure 3E,F).

Gap junctions between CCs and oocytes are crucial for transferring essential metabolites, including ROS scavengers necessary for oocyte function [31]. NADPH is critical in maintaining intracellular glutathione (GSH) levels, an antioxidant produced by CCs and supplied to oocytes. We observed significantly reduced GSH levels in OEM oocytes (Figure 3G,H), indicating an impaired antioxidant defence. DCFH staining revealed elevated ROS levels in OEM oocytes (Figure 3I,J), accompanied by reduced ATP production, signalling mitochondrial dysfunction (Figure 3L). Analysis of mitochondrial membrane potential (MMP) using JC‐1 staining showed significantly lower MMP in OEM oocytes compared to Sham controls, suggesting compromised mitochondrial activity (Figure 3K,M).

Excess ROS and disrupted energy metabolism can lead to oxidative DNA damage and increased apoptosis [33]. We found elevated DNA damage in OEM oocytes using γ‐H2A.X staining (Figure 3N,O). Annexin‐V staining revealed nearly a threefold increase in early oocyte apoptosis in OEM mice (30.61 ± 6.714) compared to Sham controls (10.17 ± 2.660) (Figure 3P,Q). These findings indicate that OEM negatively affects oocyte viability, likely due to compromised CC function and disrupted antioxidant supply through TZPs, underscoring the detrimental effects of OEM on oocyte quality.

### 3.4 OEM Induces Meiotic Arrest, Mitochondrial Dysfunction, and Spindle Abnormalities in Oocytes

**FIGURE 4:**
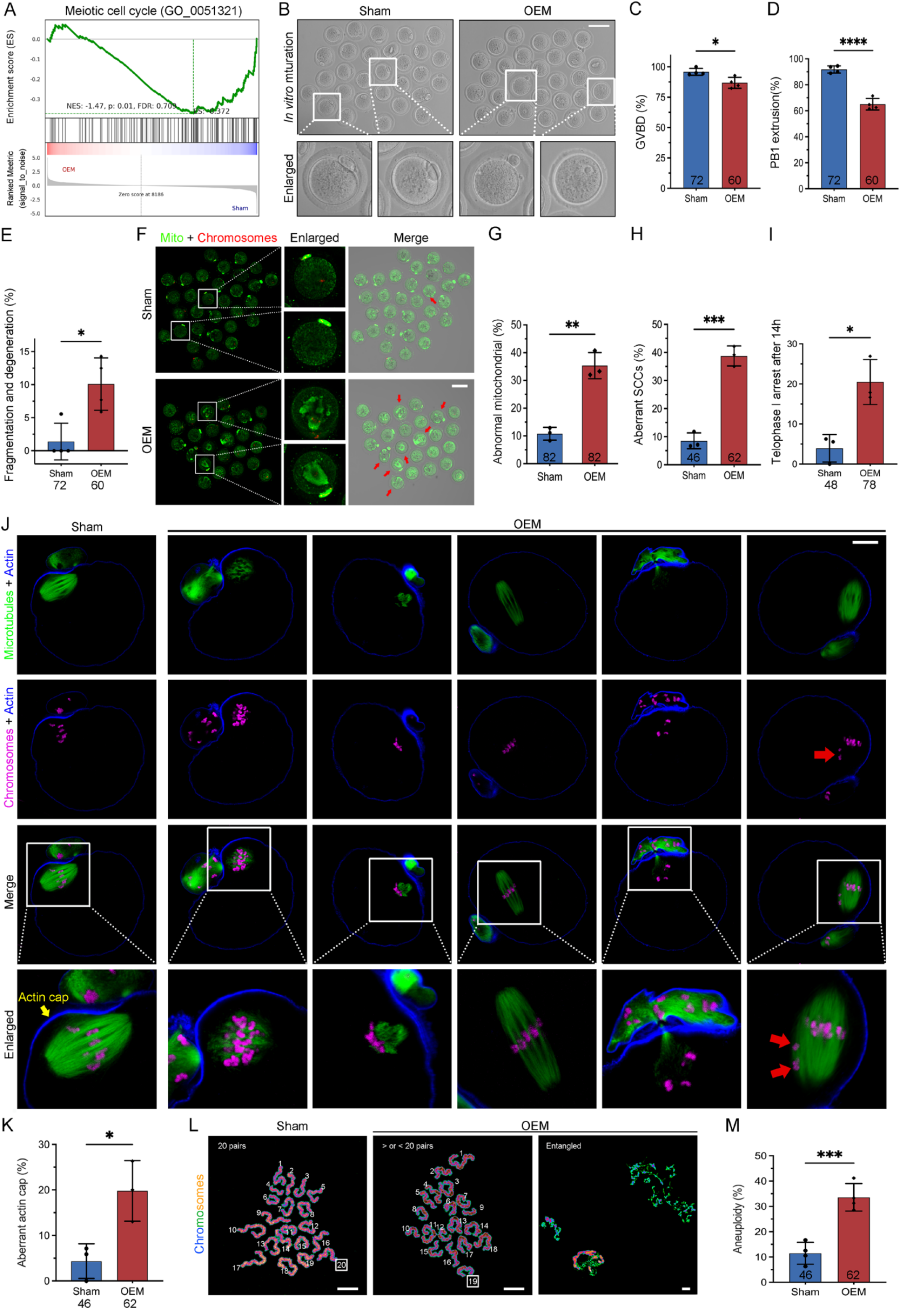
Maturation arrest, mitochondrial dysfunction, and meiotic defects induced by OEM. (A) GSEA showing enrichment of genes in the “meiotic cell cycle” pathway in OEM ovaries. (B) Representative images of MII oocytes after in vitro maturation (Scale bar, 100 μm). (C–E) Quantification of GVBD, PB1 emission, and fragmentation and degeneration of oocytes after in vitro culture. Sham oocytes, *n* = 72; OEM oocytes, *n* = 60. (F) MitoTracker Green and Hoechst 33342 staining of mitochondrial localization (green) and DNA (red). (G) Quantification of oocytes with clustering mitochondrial distribution. Sham oocytes, *n* = 82; OEM oocytes, *n* = 82. (H) Abnormal spindle assembly in MII oocytes and (I) telophase I arrest after 14 h of in vitro maturation. (J) Confocal microscopy images show spindle assembly and PB1 emission. Microtubules (green), chromosomes (magenta), and F‐actin (blue) are stained (Scale bars, 25 μm). (K) Quantification of MII oocytes with aberrant actin caps. Sham oocytes, *n* = 46; OEM oocytes, *n* = 62. (L) Chromosome spread images with numbers of paired sister chromatids (Scale bar, 5 μm). (M) Aneuploidy rates in oocytes collected after in vitro maturation. Sham oocytes, *n* = 46; OEM oocytes, *n* = 62. Statistical significance was set at *p < 0.05, **p < 0.01, ***p < 0.001, and ****p < 0.0001. Not significant (ns) at *p* ≥ 0.05.

RNA‐seq analysis of ovaries from Sham and OEM mice revealed a downregulation of key genes involved in oocyte meiosis and maturation (Figure 4A), indicating compromised oocyte development in the presence of OEM. High oxidative stress and early apoptosis in GV‐stage oocytes from OEM mice further indicated their reduced maturation potential, highlighting the impact of OEM on oocyte quality. In vitro culture of fully grown GV oocytes from OEM mice showed a slower rate of germinal vesicle breakdown (GVBD) (Figure 4B,C) and a significantly delayed emission of polar body‐1 (PB1) compared to Sham controls (Sham: 91.77 ± 2.739, OEM: 65.04 ± 4.459) (Figure 4D). Additionally, OEM oocytes exhibited a significant increase in fragmentation and degeneration (Figure 4E).

Mitochondrial dysfunction, indicated by abnormal distribution patterns, was significantly more prevalent in mature oocytes from OEM mice compared to controls (Sham: 10.78% ± 2.3%, OEM: 35.36% ± 4.7%) (Figure 4F,G), suggesting impaired cytoplasmic maturation. This was accompanied by meiotic arrest, primarily due to defective spindle organisation, a critical factor for successful fertilisation. Metaphase II (MII)‐stage oocytes from OEM mice exhibited a higher incidence of spindle abnormalities, including disordered, unipolar, and elongated spindles, and misaligned chromosomes that failed to assemble at the equatorial plate (Sham: 8.525 ± 2.816, OEM: 38.75 ± 3.568) (Figure 4H,J). An increase in oocytes arrested in the anaphase I and telophase I (AT1) phases was also observed in OEM mice (Figure 4I).

Despite appearing to release PB1, many OEM oocytes remained at meiosis I telophase with incomplete cytokinesis, where PB1 remained attached to the oocyte via a cytoplasmic bridge, and MII spindles failed to assemble fully. Actin staining further revealed disrupted actin caps in a higher proportion of OEM oocytes following in vitro maturation (Figure 4J,K). Chromosome spread analysis showed elevated aneuploidy rates in OEM oocytes, with entangled and unseparated chromosome masses and chromatid pair deviations (33.58%), compared to Sham oocytes, which predominantly showed the normal 20 pairs of sister chromatids (88.51%) (Figure 4L,M). These findings indicate that OEM leads to maturation arrest, mitochondrial dysfunction, and meiotic defects, significantly impairing oocyte quality and developmental competence.

### 3.5 Melatonin Enhances Oocyte Quality and Oocyte‐Cumulus Cell Communication in OEM

**FIGURE 5:**
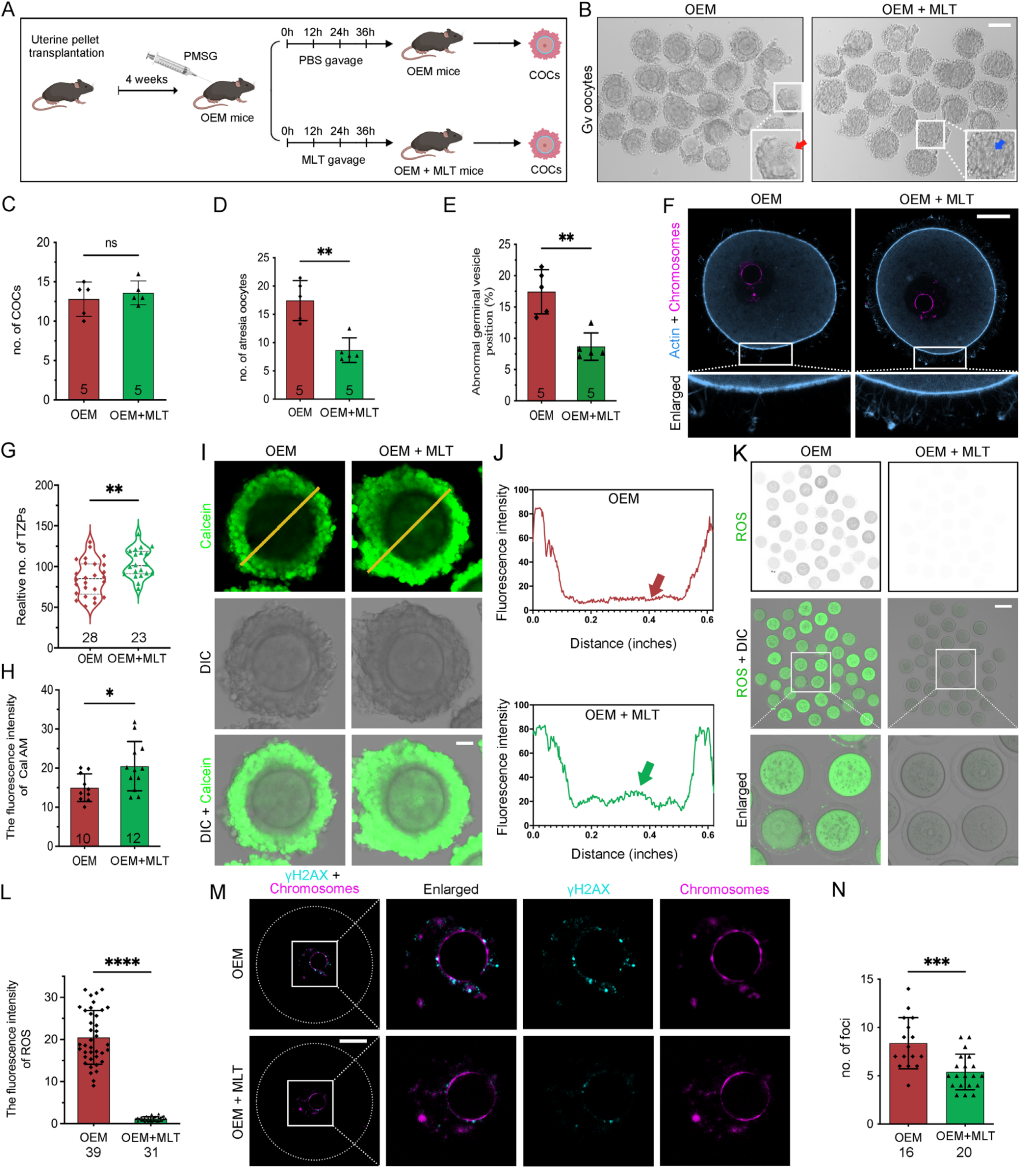
Melatonin restores oocyte‐granulosa cell communication and quality in OEM mice. (A) Experimental timeline depicting melatonin (MLT) administration and hormone injection for superovulation. (B) Representative images of COCs in OEM and OEM + MLT mice (Scale bar, 120 μm). (C, D) Quantification of COCs and atretic oocytes in OEM and OEM + MLT mice. OEM mice, *n* = 5; OEM + MLT mice, *n* = 5. (E) Recording of peripheral GV rate in OEM and OEM + MLT oocytes. OEM mice, *n* = 5; OEM + MLT mice, *n* = 5. (F) Confocal images of COCs from OEM and OEM + MLT mice with TZPs (blue) labelled by phalloidin. Magenta outlines indicate the oocyte nucleus. (G) Quantification of TZP number in confocal sections. OEM oocytes, *n* = 28; OEM + MLT oocytes, *n* = 23. (H) IOD measurement reflecting gap junction permeability. OEM oocytes, *n* = 10; OEM + MLT oocytes, *n* = 12. (I, J) Calcein dye transmission and corresponding fluorescence intensity distributions (Scale bar, 25 μm). (K, L) DCFHDA staining and ROS fluorescence intensity quantification (Scale bar, 100 μm). OEM oocytes, *n* = 39; OEM + MLT oocytes, *n* = 31. (M, N) γ‐H2AX staining and quantification of DNA damage in OEM and OEM + MLT oocytes (Scale bar, 25 μm). OEM oocytes, *n* = 16; OEM + MLT oocytes, *n* = 20. Statistical significance was set at *p < 0.05, **p < 0.01, ***p < 0.001, and ****p < 0.0001. Not significant (ns) at *p* ≥ 0.05.

Our findings revealed that OEM reduces oocyte‐CC communication by damaging TZPs, leading to oxidative stress, mitochondrial dysfunction, and impaired oocyte maturation. Given melatonin's (MLT) established role in alleviating EM progression and stimulating TZPs and actin production [12, 34, 35], we explored its potential to enhance oocyte quality and improve oocyte‐CC communication in OEM mice (Figure 5A). Mice received MLT via gavage (30 mg/kg body weight) at 0, 12, 24, and 36 h post‐PMSG superovulation, while controls were given PBS. Although the MLT‐treated group showed a modest, non‐significant increase in oocyte numbers, the number of atretic oocytes significantly decreased compared to controls (Figure 5B–D). Additionally, MLT improved GV‐stage oocytes morphology (Figure 5E).

Immunofluorescence analysis revealed a significant increase in TZP numbers following MLT treatment, suggesting improved oocyte‐CC connectivity (Figure 5F,G). Enhanced calcein‐AM fluorescence in the MLT group further indicated improved gap junction functionality (Figure 5H–J). Furthermore, MLT significantly decreased oocyte oxidative stress levels, reducing them approximately 17‐fold, from 20.49 ± 6.381 in OEM mice to 1.155 ± 0.4915 in the MLT‐treated group (Figure 5K,L). MLT administration also ameliorated DNA damage in oocytes, as indicated by reduced γ‐H2A.X staining (Figure 5M,N). These findings suggest that MLT improves oocyte quality in OEM mice by enhancing oocyte‐CC communication, reducing oxidative stress, and minimising DNA damage.

### 3.6 In Vitro Melatonin Supplementation Enhances Oocyte Quality in OEM Mice

**FIGURE 6:**
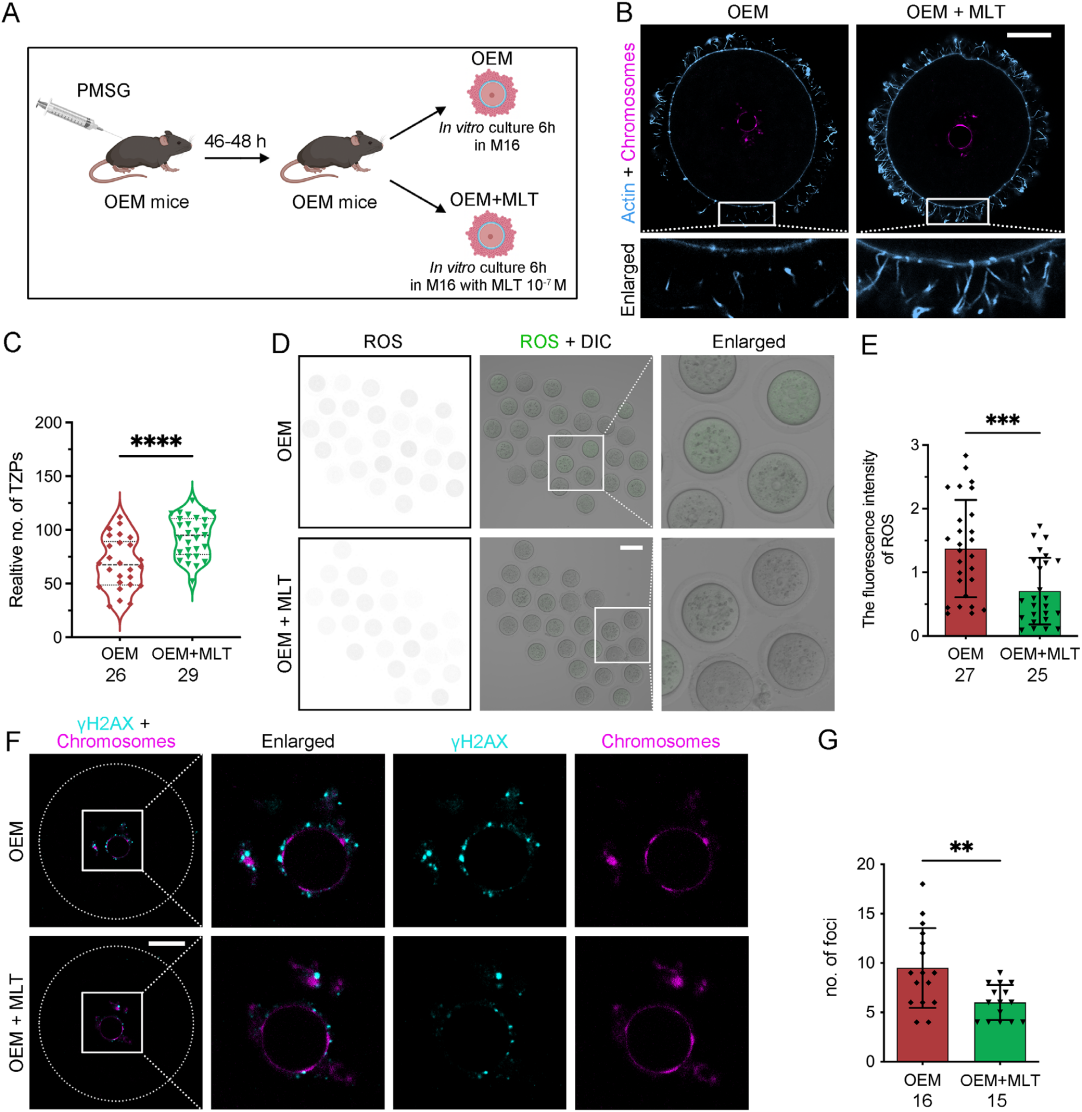
Melatonin supplementation in vitro inhibits TZP retraction and mitigates oxidative stress in OEM mice. (A) Experimental timeline for in vitro MLT supplementation. (B) Confocal images of TZPs (blue) in COCs from OEM and OEM + MLT mice. (C) TZP number quantification in equatorial confocal sections. OEM oocytes, *n* = 26; OEM + MLT oocytes, *n* = 29. (D) DCFHDA staining of ROS in OEM and OEM + MLT oocytes (Scale bar, 100 μm). (E) Quantification of ROS fluorescence intensity. OEM oocytes, *n* = 27; OEM + MLT oocytes, *n* = 25. (F, G) γ‐H2AX staining and quantification of DNA damage in OEM and OEM + MLT oocytes (Scale bar, 25 μm). OEM oocytes, *n* = 16; OEM + MLT oocytes, *n* = 15. Statistical significance was set at *p < 0.05, **p < 0.01, ***p < 0.001, and ****p < 0.0001. Not significant (ns) at *p* ≥ 0.05.

To explore more effective options for ART of OEM patients, we performed superovulation on OEM mice and supplemented the culture medium with MLT (1 × 10^−7^ mol/L) for 6 h to assess its impact on oocyte quality (Figure 6A). Like the effects observed with oral MLT administration, in vitro MLT supplementation prevented the retraction of TZPs, thereby preserving oocyte‐CC communication (Figure 6B,C). Furthermore, MLT significantly reduced oxidative stress levels in the oocytes (Figure 6D,E) and minimised DNA damage associated with OEM (Figure 6F,G). These findings indicate that in vitro MLT supplementation effectively improves oocyte quality in OEM, supporting its potential use as an adjunct treatment in ART for OEM patients.

### 3.7 Melatonin Supplementation In Vitro Enhances Meiotic Maturation, Reduces Oxidative Stress, and Mitigates Aneuploidy in OEM Mice

**FIGURE 7:**
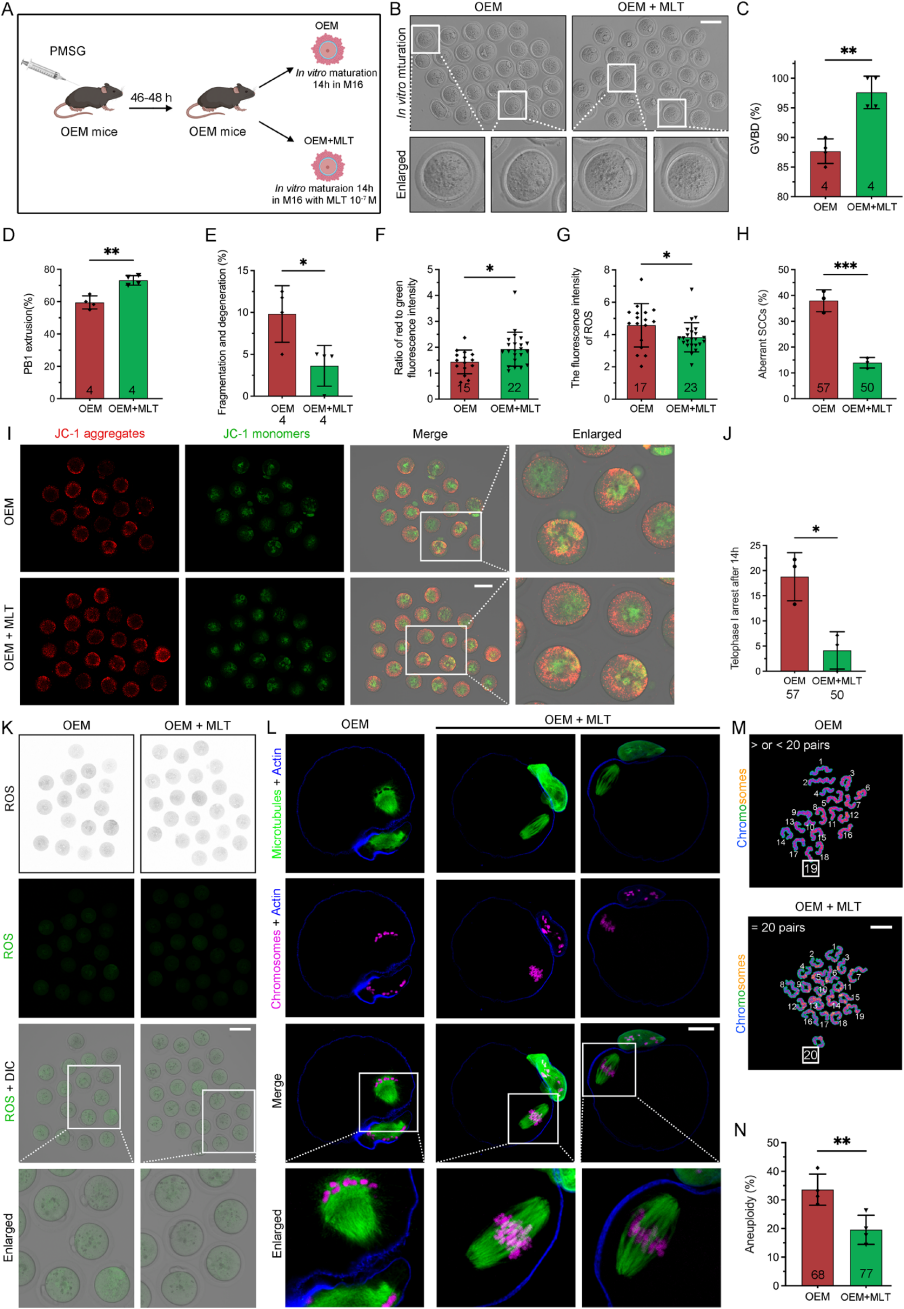
Melatonin supplementation in vitro rescues meiotic maturation in OEM oocytes. (A) Timeline diagram illustrating MLT addition during in vitro maturation. (B) Representative images of oocytes after in vitro maturation with M16 (Scale bar, 100 μm). (C–E) Rates of GVBD, PB1 emission, and oocytes fragmentation and degeneration after in vitro culture. OEM mice, *n* = 4; OEM + MLT mice, *n* = 4. (F) JC‐1 intensity ratio of aggregates to monomers in oocytes, quantified via ImageJ. OEM oocytes, *n* = 15; OEM + MLT oocytes, *n* = 22. (G) ROS fluorescence intensity in OEM (*n* = 17) and OEM + MLT (*n* = 23) oocytes. (H) Abnormal spindle assembly rates in OEM (*n* = 57) and OEM + MLT (*n* = 50) oocytes. (I) JC‐1 monomer and aggregate staining of oocytes. (J) Rates of telophase I arrest after 14 h of in vitro maturation. OEM oocytes, *n* = 57; OEM + MLT oocytes, *n* = 50. (K) DCFHDA staining of ROS levels in oocytes (Scale bar, 100 μm). (L) Confocal images show spindle assembly and PB1 emission (Scale bars, 25 μm). (M, N) Chromosome spread and aneuploidy rates in oocytes post‐maturation (Scale bar, 5 μm). Statistical significance was set at *p < 0.05, **p < 0.01, ***p < 0.001, and ****p < 0.0001. Not significant (ns) at *p* ≥ 0.05.

We investigated the effect of MLT supplementation in vitro during oocyte maturation in OEM mice (Figure 7A). The GVBD rate significantly increased from 87.68% ± 2.074% in the OEM group to 97.62% ± 2.749% in the MLT‐treated group (Figure 7B,C). Similarly, the emission rate of PB1 improved from 59.51% ± 4.040% in OEM oocytes to 73.15% ± 2.918% following MLT treatment (Figure 7D). MLT also reduced the proportion of fragmented and degenerated oocytes from 9.816% ± 3.378% (OEM) to 3.631% ± 2.423% (OEM + MLT) (Figure 7E).

MLT treatment significantly enhanced MMP levels, as indicated by JC1 staining, demonstrating improved cytoplasmic maturation in oocytes (Figure 7F,I). MLT also reduced oxidative stress levels (Figure 7G,K) and ameliorated abnormalities in spindle‐chromosome complexes (SCCs) in mature oocytes from OEM mice (Figure 7H,L). Notably, the incidence of AT1 abnormalities reduced from 18.80% ± 4.782% in OEM oocytes to 4.135% ± 3.703% in the MLT‐treated group (Figure 7J). Chromosome spreads and Hoechst staining further demonstrated that MLT significantly reduced oocyte aneuploidy associated with OEM (Figure 7M,N). In summary, MLT supplementation in vitro improved oocyte quality by enhancing meiotic maturation, reducing oxidative stress, and mitigating the effects of aneuploidy and disrupted oocyte‐cumulus cell communication in the OEM mouse model.

## 4 Discussion

Ovarian endometriosis (OEM) is a common gynaecological condition that significantly impacts reproductive health but remains inadequately researched. This study provides new insights into how OEM impairs fertility by disrupting the critical communication between oocytes and cumulus cells (CCs), resulting in oxidative stress and mitochondrial dysfunction. Our findings highlight the role of melatonin (MLT) as a therapeutic agent that can mitigate these deleterious effects, offering a potential intervention to restore reproductive function in OEM patients.

Transzonal projections (TZPs) are essential cytoplasmic projections that connect CCs to the oocyte through the zona pellucida, facilitating the transfer of metabolites, RNA, and signalling factors crucial for oocyte development [36, 37, 38, 39, 40, 41]. In EM patients, the integrity of gap junctions between granulosa cells may be compromised [42]. Our study confirms that OEM induces a significant reduction in TZPs, leading to impaired oocyte‐CC communication. This disruption is accompanied by a downregulation of critical regulatory genes such as *Cyp19a1* and FSHr, which are essential for maintaining follicular development and hormone synthesis. These changes impair ovarian function, leading to epigenetic modifications and disrupted oestrogen receptor signalling, adversely affecting oocyte and embryo quality [43]. The epigenetic integrity of oocytes is crucial for successful embryonic development [44], as even oocytes fertilised with hypomethylated sperm can exhibit normal developmental potential if their epigenetic structure is intact [45, 46].

The importance of maintaining TZP integrity is underscored by its role in sustaining a healthy follicular environment. Our findings align with previous research demonstrating that EM induces iron overload and oxidative damage in granulosa cells via the ROS/HIF‐1α/FSHr signalling pathway [47, 48]. Specifically, the downregulation of FSHr observed in our study likely delays follicular response, impairs dominant follicle formation, increases atretic follicle rates, and compromises TZP formation [49, 50]. Moreover, abnormal germinal vesicle (GV) positioning in OEM oocytes indicates disrupted oocyte polarity, a hallmark of reduced oocyte quality, which correlates with compromised FSH signalling [49, 51]. These findings provide a more nuanced understanding of the molecular mechanisms by which OEM impairs oocyte function.

Oxidative stress and mitochondrial dysfunction are well‐established features of EM [4, 32], contributing to apoptosis and reduced glutathione (GSH) metabolism in granulosa cells [12]. In normal conditions, CCs metabolise glucose and supply pyruvate to support oocyte ATP production [52, 53], while GSH produced by CCs is critical for scavenging ROS in oocytes. However, in OEM mice, the breakdown of oocyte‐CC communication disrupts this antioxidant and energy substrate transfer, resulting in decreased GSH levels and exacerbated oxidative stress in oocytes. This disruption compromises oocyte quality, as evidenced by increased DNA oxidation and reduced ATP levels, which ultimately hinder oocyte maturation.

Our study further supports previous findings that elevated pyruvate levels in the follicular fluid of EM patients may be indicative of impaired oocyte‐CC communication [54]. Additionally, our RNA sequencing data revealed upregulation of *Cyp2e1*, a gene associated with ROS production, suggesting a potentially overlooked mechanism by which oxidative stress exacerbates ovarian damage in OEM [55, 56]. The disruption of redox balance in oocytes leads to oxidative stress, meiotic arrest, and spindle disorganisation, further compromising oocyte quality. Moreover, the observed loss of the actin cap and abnormal GV positioning in OEM oocytes indicate compromised oocyte polarity, reduced maternal DNA protection, potentially increasing aneuploidy risk [57]. Clinical studies have shown elevated rates of embryo aneuploidy in EM patients [58], underscoring the need for careful selection of GV oocytes during ART to improve maturation and fertilisation outcomes. This also suggests that improving sperm entry points during intracytoplasmic sperm injection (ICSI) may reduce the risk of heteroploidy [59].

Current treatments for EM focus on pain management and fertility improvement [60], yet reproductive function often remains compromised post‐treatment. Our study demonstrates that both oral and in vitro MLT administration mitigates the deleterious effects of OEM on oocyte‐CC communication. MLT treatment restores TZP integrity, enhances mitochondrial function, and reduces oxidative stress, thereby improving oocyte maturation. These results align with previous research showing that MLT reduces oxidative stress‐induced senescence in granulosa cells and supports early embryo development by promoting microfilament and filopodia production [12, 34, 35, 61]. Moreover, MLT has been shown to improve IVF outcomes in older women and relieve pain in EM patients [25, 62]. Abnormal sleep rhythm, especially staying up late, can lead to elevated prolactin levels, which are a major contributor to pain in EM patients. MLT improves sleep quality and likely regulates prolactin fluctuations, thereby reducing the pain associated with them [63]. Thus, MLT offers a dual therapeutic benefit by addressing both the physiological and psychological aspects of EM.

While our study demonstrates MLT's effectiveness in mitigating OEM‐induced damage, future clinical trials are necessary to assess its efficacy in human patients. Additionally, our findings underscore the need for targeted therapeutic interventions aimed at preserving oocyte quality, particularly in patients undergoing ART. As previous studies have shown no significant differences in IVF outcomes between OEM patients who underwent conservative ovarian surgery and those who proceeded directly to IVF [64], the potential for MLT to enhance IVF success in OEM patients warrants further exploration.

Given the challenges of obtaining human ovarian samples, we utilised a mouse model replicating key OEM features, along with a peritoneal model for comparison. While no significant differences in oocyte maturation were observed in the peritoneal model after 1 month (data not shown), the OEM model revealed more pronounced effects on ovarian function [65]. The mouse ovarian bursa isolates the ovaries from the peritoneal cavity [66], suggesting its role in shielding developing follicles and emphasising the importance of considering its removal when using the peritoneal model in ovarian research.

While ART is commonly used for infertility in EM patients, challenges such as reduced oocyte numbers and lower fertilisation rates persist [4, 67]. The variability in outcomes may depend on EM subtype [26, 68, 69]. Our findings indicate a decrease in TZP numbers around most oocytes in OEM mice, though viable oocytes capable of fertilisation can still be obtained. Future research should focus on large‐scale clinical trials to evaluate MLT's therapeutic potential in human patients, as well as the development of advanced screening tools to assess oocyte quality based on TZP integrity and mitochondrial function.

In conclusion, unravelling the transcriptomic and molecular mechanisms of OEM‐induced cytophysiopathological changes, along with the potential reversibility of these effects via MLT in murine COCs, provides valuable insights for improving the selection of high‐quality nuclear recipient oocytes in ART. This is especially crucial for females with OEM or OEM‐like symptoms. Additionally, research on preserving TZP integrity, along with advances in technologies like remodelling of ovarian follicles and oocyte nuclear transplantation [70, 71], holds great promise for improving fertility outcomes [72, 73, 74]. Given the pivotal role of TZPs in oocyte function, these innovations could lead to new therapeutic strategies aimed at restoring oocyte quality in conditions such as OEM.

## Author Contributions

L.G., Y.Y. and Y.G. contributed equally to this work; J.V.Z., L.G. and M.Y. designed the conception of this work; L.G., Y.Y., Y.G., T.X., H.W., J.C. and M.L. performed the research; L.G. and J.V.Z. analysed the data; T.X. managed projects; L.G., Y.G. and J.V.Z. wrote the manuscript. L.G., Y.G., J.V.Z. and M.Y. revised the figures and manuscript. J.V.Z., P.J., M.Y. and W.C. performed supervision, acquired resources and funding. All authors approved the final version of the manuscript.

## Conflicts of Interest

The authors declare no conflicts of interest.

## Data Availability

The data that support the findings of this study are available from the corresponding author upon reasonable request.
